# Child Maltreatment in Western China: Demographic Differences and Associations with Mental Health

**DOI:** 10.3390/ijerph16193619

**Published:** 2019-09-26

**Authors:** Yueyue Zhou, Yiming Liang, Jin Cheng, Hao Zheng, Zhengkui Liu

**Affiliations:** 1 CAS Key Laboratory of Mental Health, Institute of Psychology, Beijing 100101, China; zhouyueyue@psych.ac.cn (Y.Z.); liangym@psych.ac.cn (Y.L.); zhengh@psych.ac.cn (H.Z.); 2 Department of Psychology, University of Chinese Academy of Sciences, Beijing 100049, China; 3 School of Psychology, Beijing Sport University, Beijing 100084, China; chengjin_18@outlook.com

**Keywords:** child maltreatment, neglect, depression, anxiety, China

## Abstract

*Background:* Child maltreatment has become a serious public health and social problem worldwide. However, knowledge regarding the status of child maltreatment in western China is limited. *Objective:* The objective of this study was to investigate the status of child maltreatment in western China and its relationship with mental health. *Participants and setting:* The present study evaluated child maltreatment in a sample of 1511 children (*M*_age_ = 11.48 years) from western China. *Methods:* The participants completed questionnaires designed to collect demographic information and assess their experiences with maltreatment and symptoms of depression and anxiety. *Results:* In total, 12.3%, 14.0%, 1.3% and 28.1% of the children experienced physical abuse, emotional abuse, sexual abuse and neglect, respectively, while 186 children (12.3%) experienced multiple types of maltreatment. Boys were more likely to experience maltreatment than girls in most cases. Physical abuse, emotional abuse, sexual abuse and neglect had unique effects on depression symptoms. Physical abuse, emotional abuse and neglect, but not sexual abuse, had unique effects on anxiety symptoms. *Conclusions:* The children who experienced maltreatment had higher levels of depressive and anxiety symptoms. Population-based prevention and educational programs should highlight the serious negative effects of maltreatment, especially emotional abuse and neglect, which have long been ignored in China.

## 1. Introduction

According to the United Nations Convention on the Rights of the Child, children are entitled to basic rights of protection, including protection from abuse, neglect, exploitation, and violence [1]. However, this right faces serious challenges, as child maltreatment has become a serious public health and social problem worldwide [2,3,4]. Child maltreatment refers to “all forms of physical and/or emotional ill-treatment, sexual abuse, neglect or negligent treatment, or commercial or other exploitation of children that results in actual or potential harm to a child’s health, survival, development, or dignity” [5] (p. 59). In general, there are four forms of child maltreatment: physical abuse, emotional abuse, sexual abuse and neglect [2]. To date, researchers have conducted numerous studies focusing mainly on the prevalence, types, risk and protective factors and adverse effects of child maltreatment [4,6].

Because of differences in measurement approaches and samples, the prevalence of child maltreatment varies across studies. Stoltenborgh and colleagues conducted a meta-analysis to estimate the global prevalence of child maltreatment and found that 22.6%, 36.3%, 12.7% and 17.4% of children have experienced physical abuse, emotional abuse, sexual abuse and neglect, respectively [4]. Even worse, many children have been subjected to multiple types of maltreatment [7,8]. For example, Nguyen and colleagues found that 20.7%, 14.5% and 6.3% of 15-year-old Vietnamese children have experienced two, three and all four types of maltreatment, respectively [7]. However, no single study investigated the status of multiple maltreatment in Chinese children.

Given the relatively high prevalence of child maltreatment, it is imperative to identify children who are vulnerable to maltreatment. Some studies focused on demographic risk factors such as sex, only child status and parents’ education level [6,9,10]. In these studies, researchers extensively examined sex differences in maltreatment, but the findings were mixed. For example, some studies found a higher percentage of sexual abuse among boys [6,11], whereas others found a higher percentage among girls [4,12]; still other studies found no sex differences [7]. The same results regarding sex were reported for the other three types of maltreatment. Due to differences in measurement approaches, samples and culture across studies, it is difficult to speculate on the reasons for these sex differences. Parental education level also attracted considerable attention, but the results for this variable were also mixed. For example, Leung and colleagues found that a mother’s low education level was associated with higher levels of physical abuse [11]. In addition, in a sample of 17-year-old adolescents, Chen and colleagues found no statistically significant associations between the experience of sexual abuse and the father’s or mother’s education level [9]. Moreover, the authors found no statistically significant relations between experiencing sexual abuse and whether the child lived in a single- or multiple-child family [9]. More research is needed to examine which subgroups of children are more vulnerable to maltreatment.

More importantly, child maltreatment has both short- and long-term deleterious impacts on children’s mental health [3]; for example, it can increase the risk of depression, anxiety, aggression and risky sexual behavior [3,13]. Among these, depression and anxiety, as the major burdens of mental illness [14], are two of the most frequently studied mental health problems that result from maltreatment [15,16]. For example, a recent meta-analysis demonstrated that more than half of adult depression and anxiety cases worldwide are caused by childhood maltreatment [16]. Preventing the incidence of maltreatment may be an effective way to decrease depression and anxiety cases [16].

However, an open question remains regarding whether some types of maltreatment (e.g., sexual and physical abuse) are more harmful than others. Exploring this issue, a longitudinal study lasting 27 years found that while sexual abuse had no effect, the other three forms of maltreatment had equivalent effects on depression and anxiety [13]. Contrary to this finding, a recent meta-analysis found that sexual and physical abuse had the greatest impacts on depression and anxiety disorders [16]. More concretely, individuals with a history of physical abuse, sexual abuse, or neglect were 2.00, 2.66, and 1.74 times more likely, respectively, to have depression or anxiety disorders in adulthood than those without such a history.

While previous studies yielded fruitful findings on child maltreatment, few studies have been carried out in Asian countries, especially China. In fact, studies conducted in non-Asian cultural contexts cannot be generalized to China, as the status of child maltreatment varies according to cultural background [2,7]. Thus, researchers have called for more research in this area. To our knowledge, only a few empirical studies investigated childhood maltreatment in Chinese samples [9,11,17,18,19,20,21,22,23,24]. Chen and colleagues initiated a study investigating sexual abuse in mainland China [9,18]. In a population of senior high school students, they found that the prevalence of unwanted sexual experience among boys and girls was 10.5% and 16.7%, respectively. Furthermore, 8.9% of girls and 5.0% of boys had experienced at least one type of physical-contact sexual abuse. Finally, only 1% of adolescents had experienced sexual penetration before 16 years of age. In addition to studying the prevalence of sexual abuse, the authors found that compared with adolescents without sexual abuse experience, those with sexual abuse experience had higher rates of depression [18]. Leung and colleagues assessed physical abuse and sexual abuse in a large sample of adolescents (mean age of 14.68 years) in Guangzhou City, China. The results indicated that in the previous six months, 23.2%, 15.1%, and 2.8% of the adolescents had experienced minor, severe, and very severe physical abuse, respectively. However, the prevalence of sexual abuse was only 0.6% [11,22].

Although these studies conducted in China provided abundant information, several gaps remain in the empirical literature. First, the literature in this field has been dominated by research on sexual abuse, followed by physical abuse, whereas emotional abuse and neglect have generally been ignored. Thus, far, few studies have investigated the four types of maltreatment in a general sample of children. Thus, the prevalence of different types of maltreatment remains unknown, as does how many different combinations of maltreatment types exist among Chinese children. Second, some studies assessed childhood maltreatment by relying only on adult/adolescent retrospective reporting, which may have introduced recall bias [19,23] and further led to unreliable measurements of maltreatment [4]. Finally, although previous studies investigated the impact of maltreatment on mental health, no studies have examined the specific effects of each form of maltreatment on mental health in a Chinese sample. Thus, whether all forms of maltreatment have the same effects on mental health remains unknown.

In general, child maltreatment is culturally dependent, such that its prevalence, risk factors, and impact on mental health vary across cultures. In China, especially in western China, research on child maltreatment is still scarce. To fill these gaps, the aims of the present study were to (i) describe the prevalence of child maltreatment by sex in a relatively large sample of children from western China; (ii) examine the possible demographic risk factors of maltreatment; and (iii) examine the unique contributions of the four types of maltreatment to depression and anxiety.

## 2. Method

### 2.1. Participants

The data for the present study came from a large survey conducted by World Vision International, a nongovernmental organization with the aim of improving the lives of children in underprivileged areas. The present survey was designed to explore the status of child maltreatment and child protection in western China. Children were recruited from four counties/cities in Northwest and Southwest China using cluster random sampling. Two counties in Shanxi Province and two counties in Sichuan Province were included. A total of 1511 children from 30 primary schools participated in the survey. The mean age of the children was 11.48 years (*SD* = 0.59, range = 10–15 years). Of the 1511 children, 806 (53.3%) were boys, and 705 (46.7%) were girls.

Approximately 1/3 of the participants (33.8%) were the only children in their family. Most children (81.0%) lived at home on school days, whereas 19.0% lived at school. Parents were the primary caregivers for 77.6% of the children, while grandparents, brothers, sisters and others were the primary caregivers for the remaining children. Approximately 62.5% of the children lived with both parents, whereas 37.5% were left-behind children (by one or both parents). In terms of parental education, approximately 51.5% of fathers and 54.9% of mothers had completed a junior high school degree or below, and 48.5% of fathers and 45.1% of mothers had completed a senior high school degree or above. This sample is representative of the situation in western China.

### 2.2. Procedure

The large survey was conducted during September and October 2016. All the research procedures were approved by the Research Ethics Review Board of the Institute of Psychology, Chinese Academy of Sciences (ethical approval code was H16026). Written informed consent was obtained from the children, and oral consent was obtained from teachers, school administrators, parents or other primary caregivers. We did not contact the parents of all the children; in some cases, we contacted the primary caregivers, such as the grandparents, of the left-behind children whose parents did not work locally and could not be reached. Trained research assistants administered the questionnaires using a uniform procedure during regular school hours. The survey was not anonymous due to the need to identify and track the children for the follow-up investigations and the subsequent psychological interventions. We have a special non-governmental organization responsible for child protection and psychological services in this area. Its work has been recognized and supported by local governments, schools and communities. To create a safe environment, we repeatedly emphasized to the children that the survey data were for research purposes only and that their personal information would not be communicated to family members, teachers or anyone else. The children were told that their participation was completely voluntary. Finally, all the children received a notebook as a gift for participating.

### 2.3. Measures

#### 2.3.1. Maltreatment 

Consistent with some studies in this field that used one or two items to measure child maltreatment [19,25,26,27], we used one item with a yes/no response option to assess each type of maltreatment. The children were asked if they had ever experienced (1) physical abuse, such as corporal punishment; (2) emotional abuse, such as shame or intimidation; (3) sexual abuse, such as indecent assault or sexual harassment; and (4) neglect, such as with their ideas or opinions ignored. If a child answered “yes” to the corresponding question, he/she was classified as with experienced that type of maltreatment.

#### 2.3.2. Depression 

We used the 10-item Chinese short version of the Center for Epidemiologic Studies Depression Scale (CES-D) to assess depression symptoms [28,29]. The CES-D is one of the most widely used measures of depression symptoms, and its good reliability and validity in Chinese samples has been demonstrated across a wide range of age groups [30]. Based on the results of exploratory factor analysis, two items were deleted due to low factor loadings (lower than 0.35). Item examples include “I felt depressed” and “I felt that everything I did took me quite a lot of effort”. Children were asked how many days in the past week they had the following feelings or behaviors. Items were rated on a four-point scale ranging from 1 = *little or none of the time (less than one day)* to 4 = *most of the time (5 to 7 days)*. Responses across the eight items were summed, with higher scores representing higher levels of depressed mood. In the present study, the Cronbach’s alpha value for the scale was 75.

#### 2.3.3. Anxiety 

We used the 10-item Chinese short version of the State Anxiety Inventory [31,32] to assess anxiety. Item examples include “I feel nervous” and “I’m worried about what may happen”. Children were asked to assess their feelings at the moment on a four-point scale ranging from 1 = *not at all* to 4 = *very much*. The scale has been demonstrated to have good reliability and validity in samples of Chinese children [33]. Responses across the 10 items were summed, with higher scores representing higher levels of anxiety. In the present study, the Cronbach’s alpha value for this scale was 87.

#### 2.3.4. Demographic variables

Given that there may be demographic differences in child maltreatment [6,9,10], we also collected data on several important demographic variables: sex, only child status, place of residence on school days, primary caregiver, left-behind status and parents’ education level.

### 2.4. Analysis Procedure

First, we assessed how many children had experienced each type of maltreatment. Furthermore, to investigate how many different combinations of maltreatment types exist among Chinese children, we categorized the children into mutually exclusive groups and calculated the number and proportion of maltreatment by sex. Second, we used chi-square tests to compare the differences in demographic variables and *t* tests to compare levels of mental health between children exposed and not exposed to maltreatment. Next, to investigate which types of maltreatment have the greatest impact on depression and anxiety, we used a random selection method to select one type of maltreatment for each child [34]. Specifically, a random numbers table was used to randomly select one type of maltreatment for each child. For children who reported only one type of maltreatment, there was only one choice: the randomly selected maltreatment was the only type of maltreatment they had ever experienced. For children who had experienced multiple types, one type of maltreatment was randomly selected to represent their random maltreatment. At the individual level, the possibility of each maltreatment being selected was the reciprocal of the total number of types of maltreatment a child had ever experienced. We limited the assessment to only one type of maltreatment per child, because it was impossible to independently assess the effects of each type of maltreatment on mental health for those who had experienced multiple maltreatment. This method has been demonstrated to have good validity in randomly selecting trauma [35,36] and has been recommended for use in trauma research [37]. Finally, we used regression analysis to explore the unique effect of each type of maltreatment on depression and anxiety, which provided further information regarding whether different types of maltreatment have different impacts on depression and anxiety.

## 3. Results

### 3.1. Prevalence of Different Types of Child Maltreatment

In total, 186 (12.3%), 211 (14.0%), 19 (1.3%) and 425 (28.1%) of the children had experienced physical abuse, emotional abuse, sexual abuse and neglect, respectively. Regarding sex differences, a larger percentage of boys than girls had experienced physical abuse (18.11% vs. 5.67%), emotional abuse (15.88% vs. 11.77%), and sexual abuse (1.86% vs. 0.57%). No difference was observed between boys and girls with respect to neglect (28.29% and 27.94%, respectively).

Next, we calculated the number and proportion of types of maltreatment by sex. The final results are listed in Table 1. Among the 1511 participants, 608 had experienced at least one type of maltreatment, for an overall prevalence of 40.2%. A higher percentage of boys (43.8%) than girls (36.2%) had experienced maltreatment. Regarding specific combinations of types, the combination of emotional abuse and neglect was most common (*n* = 69; 4.6%). A majority of the children (*n* = 422; 27.9%) had experienced a single type of maltreatment in the past, and neglect was the most frequently reported type (*n* = 272; 18.0%).

Overall, many of the children had experienced at least one type of maltreatment. In most cases, a higher percentage of boys than girls had experienced different kinds of maltreatment. Neglect was the most common form of maltreatment.

### 3.2. Differences in Demographic Variables, Depression and Anxiety

The second aim of this study was to compare differences in demographic variables and mental health between children who had experienced maltreatment and those who had not. As shown in Table 2, a significantly larger percentage of boys than girls, children living at home rather than school, and children of mothers with an education level of senior high school or above rather than lower education levels were more likely to experience maltreatment. Only child status, primary caregiver, left-behind status and father’s education level were unrelated to maltreatment. Regarding mental health, compared with children who had never been maltreated, those who had been maltreated had higher levels of depression and anxiety.

### 3.3. Unique Contributions of the Different Types of Maltreatment to Depression and Anxiety

As shown in Table 3, all types of maltreatment had a significant positive effect on depression. The standardized regression coefficient for sexual abuse was relatively small (β = 0.06, *p* < 05), whereas neglect had the greatest impact on depression (β = 0.37, *p* < 001). Regarding anxiety, though sexual abuse did not (β = 0.02, *p* = 68), the other three types of maltreatment had a significant positive effect on anxiety, and neglect had the greatest impact (β = 0.30, *p* < 001).

Again, we conducted supplementary analyses to examine the robustness of the impact of maltreatment on mental health in two ways. First, we examined whether the results were the same when controlling for demographic variables. The results supported the robustness of our findings: the standardized regression coefficients for the four types of maltreatment changed slightly, but the significance remained unchanged. None of the covariates had significant impacts on mental health (*ps* > 05). Next, we examined whether the results were the same in the context of the non-random selection of maltreatment. For example, if a child had ever experienced both physical abuse and emotional abuse, he/she would be assigned to both the physical abuse and emotional abuse groups. The final results were generally robust as follows: the significance of most types of maltreatment remained unchanged when examining the impacts of maltreatment on mental health, except that sexual abuse had no significant impact on depression. The standardized regression coefficients for the four types of maltreatment also changed slightly.

## 4. Discussion

Child maltreatment is a serious problem worldwide and has been thoroughly studied in most parts of the world, especially North America and Europe [2,3,4]; however, few studies have revealed the current situation of child maltreatment in China. The present research is the first epidemiological study investigating multiple types of maltreatment in Chinese children.

### 4.1. Prevalence of Different Types of Child Maltreatment

We found that 186 (12.3%), 211 (14.0%), 19 (1.3%) and 425 (28.1%) children had experienced physical abuse, emotional abuse, sexual abuse and neglect, respectively. Specifically, we found that 12.3% of the children had experienced physical abuse, which is close to the prevalence reported for Asian countries (16.7%) [4] in the WHO worldwide survey (9%) [38] and in studies from Guangzhou, China [11,22]. However, the prevalence of sexual abuse was lower than that reported in meta-analyses (4.1% among boys and 11.3% among girls) and some previous studies with Chinese adolescents (10.5% among boys and 16.7% among girls) [18] but close to the WHO worldwide survey data from low-/lower-middle-income countries (1.5%) [38] and several other studies conducted in China (0.6% and 2.5%) [11,22,24]. There is little evidence regarding emotional abuse and neglect among Chinese children. Fourteen percent of the children in our sample had experienced emotional abuse, which was much lower than the findings in a meta-analysis (41.6%) [4]. Finally, the largest proportion of children (28.1%) had experienced neglect, which is close to the prevalence of emotional neglect in Asia (30.1%) [4] but much higher than the prevalence observed in the WHO worldwide survey (3.6%) [38].

In short, except for neglect, the prevalence of maltreatment in our study was close to or lower than the findings of previous studies. We should be cautious about such results and cannot conclude that the prevalence of child maltreatment is low in China. Such a seemingly low rate of child maltreatment may be correlated with Chinese culture and the characteristics of the sample. More specifically, the role of Chinese culture may affect the results in three respects. First, the Chinese have a high tolerance for maltreatment, especially nonsexual maltreatment, as illustrated by the Chinese proverb “Beating and scolding is the emblem of love” [39,40]. In traditional Chinese culture, it is acceptable to impose severe corporal punishment, verbal harm and neglect on children. Thus, even though a child is maltreated, he/she may not realize it [41]. Second, Chinese culture emphasizes children’s obedience to their parents’ authority; if children do not exhibit such obedience, they will be punished. This cultural tradition may prevent children from disclosing their experiences of maltreatment [42]. Third, children are unwilling to reveal their abuse experiences because they wish to protect their family’s reputation [42,43]. This reluctance is particularly true for sexual abuse, which is often considered a taboo subject in China, and such scandals may never become known. In addition to cultural factors, the characteristics of the sample may also contribute to the lower prevalence of sexual abuse reported by participants. For example, Chen and colleagues assessed participants’ sexual abuse experience before age 16 in a sample of 17-year-old children [9,18]. In contrast, the mean age of our sample was 11 years. As children age, the probability that they will be sexually abused greatly increases.

Consistent with the viewpoint that different types of maltreatment always co-occur [44,45], we found that many of the children had experienced multiple types of maltreatment. Among the 1511 children, 3 (0.2%), 41 (2.7%), and 142 (9.4%) had experienced four, three and two types of maltreatment, respectively. Given that it makes greater sense to compare differences in samples from the same cultural background, we call for more research to be conducted in China.

### 4.2. Demographic Differences in Child Maltreatment

To better identify groups at high risk of maltreatment, we examined demographic differences in child maltreatment. Regarding sex differences, our results (reported in Section 3.1.) indicated that in general, a significantly higher percentage of boys than girls had experienced physical abuse, emotional abuse, and sexual abuse, whereas there was no difference in neglect. These results are consistent with some previous findings [2,3,11]. For example, boys are more likely to experience physical abuse [2,3,7], emotional abuse [2,3] and sexual abuse [11]. However, our findings are also inconsistent with some studies that found that girls experience more physical abuse [4], emotional abuse [4,7,12] and sexual abuse [12]. Sex differences in neglect were also found to be mixed in previous studies [3,12]. The reasons behind these differences are complex and may be related to culture, the measurement approach and the sample. More research is needed in China to reveal the real situation and potential reasons for sex differences in child maltreatment. We believe that the boys in our study reported more sexual abuse because they dared to divulge their real experience [11], whereas the girls were less likely to disclose their experiences. In fact, the prevalence of sexual abuse among girls is significantly lower in Asian countries than in other countries [4] because society as a whole strongly emphasizes female chastity.

In addition, children living at home on school days were more likely to experience maltreatment. The reason may be that children living at home have more opportunities to experience maltreatment. A counterintuitive finding is that children of mothers with a senior high school and above level of education were more likely to experience any type of maltreatment. This finding differs from the results of previous research conducted in China [9,11]. Chen and colleagues found that parental education level was not related to sexual abuse [9], while Leung and colleagues reported that a lower maternal education level was associated with a higher likelihood of child physical abuse. To better explain our study findings, we conducted further analysis to identify which type of maltreatment was most common among the children of highly educated mothers. The results indicated that the higher the mother’s educational level, the more likely they were to neglect their children. This neglect may be caused by the double burden of work and family care. In traditional Chinese culture, women do the housework and take care of their children. However, with the development of society, an increasing number of mothers, especially those with higher education levels, go out to work. However, the tradition of women caring for the family has not changed. Therefore, mothers with higher education levels not only have to work but must also take care of their families. As a result, they do not have enough time and energy to take care of their children, which makes children feel neglected. Finally, we found no significant differences in maltreatment with respect to the following demographic variables: being an only child, the primary caregiver, left-behind status and father’s education level. More research is needed in the future to further examine the roles of these demographic variables.

### 4.3. Unique Contributions of the Different Types of Maltreatment to Depression and Anxiety

In line with previous research, our results indicated that physical abuse had a significant impact on depression and anxiety [3,15,16]. Although its adverse effect has been established, our study is the first to establish the robust effects of physical abuse on mental health by controlling for the effects of other forms of maltreatment.

We found that sexual abuse had a slight impact on depression and no impact on anxiety. Overall, our results did not strongly support the serious effects of sexual abuse. In fact, sexual abuse is the most studied [4] and most controversial form of maltreatment [13]. Our findings are consistent with some meta-analyses and recent empirical studies [13,46,47]. For example, Rind and colleagues revealed that among college students, those with childhood sexual abuse experience were slightly less well-adjusted than those without sexual abuse experience [47]. However, this difference cannot be attributed to the experience of sexual abuse but to the family environment. Of course, the serious effects of sexual abuse have been well documented [16]. Our study showed only a weak effect of sexual abuse, possibly because sexual abuse often co-occurs with other types of maltreatment, and the effect of sexual abuse was not significant after controlling for the effects of other types of maltreatment. Another reason may be that in our sample, the number of children who reported sexual abuse was relatively small, resulting in low statistical test power. Overall, we should be cautious about this result. Whether sexual abuse is serious depends on not only its nature but also its frequency and severity. Regrettably, this study did not measure its frequency and measured only less serious forms of sexual abuse. The results of our study are insufficient to conclude that sexual abuse is less harmful to mental health than other types of maltreatment. To better explore the effects of sexual abuse, future research should measure its frequency and degree of severity.

Emotional abuse has significant impacts on depression and anxiety, even after controlling for other types of maltreatment. More interestingly, child neglect, which is often ignored, had the greatest effect on both depression and anxiety. The serious effects of emotional abuse and neglect on mental health have been identified in a previous meta-analysis [3], and the adverse effects of these two forms of maltreatment outweigh even the effects of physical abuse and continue into adulthood. However, in Asian countries such as China, child maltreatment is often interpreted as physical abuse [4,7], and emotional abuse and neglect are often not considered to be child maltreatment. Therefore, parents often unconsciously emotionally abuse and neglect their children. Frequent experiences of emotional abuse and neglect result in a large cumulative effect, with extremely adverse consequences for children’s mental health. Thus, it is imperative to recognize the serious impact of emotional abuse and neglect on mental health among Chinese people.

Overall, our study further established the adverse effects of physical abuse and identified the harmful effects of emotional abuse and neglect on mental health. The impact of sexual abuse on mental health needs to be further studied in Chinese samples.

### 4.4. Strengths and Limitations

The present study has several strengths. First, this study is the first to comprehensively investigate the status of child maltreatment in western China, filling a gap in the investigation of multiple types of maltreatment in this region. Moreover, we used a random selection method to resolve the co-occurrence issue for different types of maltreatment. Some researchers have noted that the co-occurrence of maltreatment makes it difficult to identify the unique effect of each type of maltreatment [13]. Our study provides a new perspective to solve this problem in the field.

Despite the above strengths, several limitations should be noted. First, we used a single item to measure each type of maltreatment. Although it is common to measure maltreatment with one item [19,25,26], the use of a single item may lead to inaccurate measurement, which may be associated with the lower prevalence rates reported in our study. Future research should use a multi-item, behaviorally anchored measurement scale to measure all types of maltreatment. Second, we did not clearly explain the definition of maltreatment to the children before the questionnaire was administered, which may also lead to biased measurement. Third, we investigated only whether the children had experienced a specific type of maltreatment and did not assess the frequency, severity, timing of the first maltreatment or the identity of the perpetrators [12], which are equally important. For example, the adverse effect is certainly much larger for children who experience severe and prolonged physical abuse than for those who experience physical abuse only once. Fourth, although anonymous surveys are not necessarily more accurate than non-anonymous surveys [48], our survey was not anonymous, which may have affected the children’s willingness to disclose their abuse experiences. Finally, we investigated only internalizing problems, such as depression and anxiety, and did not examine externalizing problems, such as aggression. Some researchers noted that there may be a specific relationship between different types of maltreatment and adaptive outcomes [49,50]. For example, Berzenski and Yates found that emotional abuse was strongly associated with externalizing problems, such as depression and anxiety, whereas the combination of physical and emotional abuse was strongly associated with conduct-related problems, such as substance use and risky sexual behavior [49]. Although Asian children are more likely to internalize the negative effects of child maltreatment [42], only by examining internalizing and externalizing problems simultaneously can we test the specific link between different types of maltreatment and different adaptation outcomes.

### 4.5. Implications for Practice

Despite these limitations, our findings have important practical implications. First, measures, such as government-led educational programs, should be adopted to raise public awareness of child maltreatment [13], particularly emotional abuse and neglect, which have serious consequences but have long been ignored. Additionally, to protect children from maltreatment, specialized child protection agencies should be established in China. Such agencies could not only serve as protective umbrellas for maltreated children but also provide more accurate data on child maltreatment. More importantly, given that maltreatment is related to cultural characteristics, researchers and practitioners should try to develop a unique standard for maltreatment based on Chinese conditions and culture. In addition, culture-specific measurement tools for maltreatment should be developed. Especially for sexual abuse, indirect measurement is more suitable for Chinese children than direct measurement. Finally, we should pay more attention to children with the following characteristics: boys, only children, living at home and mothers with an education level of senior high school or above.

## 5. Conclusions

The present study is the first epidemiological study investigating child maltreatment in western China. We found that 12.3%, 14.0%, 1.3% and 28.1% of the children had experienced physical abuse, emotional abuse, sexual abuse and neglect, respectively, while 186 children (12.3%) had experienced multiple types of maltreatment. Boys were more likely to experience maltreatment than girls in most cases. Physical abuse (β = 0.17, *p* < 001), emotional abuse (β = 0.14, *p* < 001), sexual abuse (β = 0.06, *p* < 05) and neglect (β = 0.37, *p* < 001) had unique effects on depression symptoms. Physical abuse (β = 0.19, *p* < 001), emotional abuse (β = 0.15, *p* < 001) and neglect (β = 0.30, *p* < 001), but not sexual abuse (β = 0.02, *p* = 68), had unique effects on anxiety symptoms. Of the four subtypes of maltreatment, neglect had the greatest effect on depression and anxiety symptoms.

In summary, our study revealed the adverse effects of emotional abuse and neglect on Chinese school children. Population-based prevention and educational programs should highlight the serious harm caused by maltreatment, especially emotional abuse and neglect, which has long been ignored in China. We believe that the maltreatment we observed is only the tip of the iceberg. Researchers need to recognize that the study of child maltreatment is still in the early stages in China. Child protection has a long way to go. In any case, this study provides preliminary data on child maltreatment in China, aiming to draw the attention of Chinese researchers and the government.

## Figures and Tables

**Table 1:** Prevalence rates of different types of maltreatment.

Types of Maltreatment	Total (*n* = 1511)	Boys (*n* = 806)	Girls (*n* = 705)
	No. of Children (Percent)	No. of Children (Percent)	No. of Children (Percent)
Any type of maltreatment	608 (40.2%)	353 (43.8%)	255 (36.2%)
All four types of maltreatment	3 (0.2%)	3 (0.4%)	0 (0.0%)
Three types of maltreatment	41 (2.7%)	34 (4.2%)	7 (1.0%)
Physical abuse + emotional abuse + sexual abuse	2 (0.1%)	2 (0.2%)	0 (0.0%)
Physical abuse + emotional abuse + neglect	36 (2.4%)	29 (3.6%)	7 (1.0%)
Physical abuse + sexual abuse + neglect	2 (0.1%)	2 (0.2%)	0 (0.0%)
Emotional abuse + sexual abuse + neglect	1 (0.1%)	1 (0.1%)	0 (0.0%)
Two types of maltreatment	142 (9.4%)	87 (10.8%)	55 (7.8%)
Physical abuse + emotional abuse	29 (1.9%)	23 (2.9%)	6 (0.9%)
Physical abuse + sexual abuse	1 (0.1%)	0 (0.0%)	1 (0.1%)
Physical abuse + neglect	38 (2.5%)	27 (3.3%)	11 (1.6%)
Emotional abuse + sexual abuse	1 (0.1%)	1 (0.1%)	0 (0.0%)
Emotional abuse + neglect	69 (4.6%)	34 (4.2%)	35 (5.0%)
Sexual abuse + neglect	4 (0.3%)	2 (0.2%)	2 (0.3%)
Only one type of maltreatment	422 (27.9%)	229 (28.4%)	193 (27.4%)
Only physical abuse	75 (5.0%)	60 (7.4%)	15 (2.1%)
Only emotional abuse	70 (4.6%)	35 (4.3%)	35 (5.0%)
Only sexual abuse	5 (0.3%)	4 (0.5%)	1 (0.1%)
Only neglect	272 (18.0%)	130 (16.1%)	142 (20.1%)

**Table 2:** Comparison of demographic variables and mental health status between children who had and had not experienced child maltreatment.

Demographics	Occurrence of Abuse (*N* = 1511)		
	Yes	No	χ^2^/*t*
Sex			
Male	353	453	9.10 **
Female	255	450	
Whether the only child			
Yes	213	298	0.67
No	395	605	
Dwelling place			
Live at school	90	194	10.85 ***
Live at home	514	700	
Primary caregiver			
Parents	462	710	1.46
Others (such as grandparents, brothers and sisters)	146	193	
Whether left-behind			
Yes	241	326	1.94
No	367	577	
Father’s education level			
Junior high school or below	299	476	1.66
Senior high school or above	305	424	
Mother’s education level			
Junior high school or below	302	520	8.67 **
Senior high school or above	299	377	
Depression	13.13	10.46	13.85 ***
Anxiety	16.27	13.09	12.11 ***

Note. ** *p* < 01. *** *p* < 001. Because of missing data for some of the demographic variables, the total sample size is not equal to 1511 in some cases.

**Table 3:** Impacts of different types of maltreatment on depression and anxiety.

Predictors	Model 1 (Depression)		Model 2 (Anxiety)	
	β	*t*	β	*t*
Physical abuse	0.17	7.12 ***	0.19	7.72 ***
Emotional abuse	0.14	5.77 ***	0.15	6.19 ***
Sexual abuse	0.06	2.39 *	0.02	0.68
Neglect	0.37	14.90 ***	0.30	11.74 ***

Note. * *p* < 05. *** *p* < 001.
